# Supplementary material for: Deletion of wfs1 Impairs Oligodendrocyte Precursor Cells Dorsal Distribution and Myelination Through the wfs1-hmgcs1 Axis in Zebrafish
Source: Biology (Basel). 2026 Aug 16;15(16):1402. doi: 10.3390/biology15161402 (PMC13510151; doi:10.3390/biology15161402)
Supplement: Supplementary file 1 [file biology-15-01402-s001.zip › File S1-Transcriptome sequencing data and differentially expressed genes (DEGs) in wfs1-- zebrafish at 5 dpf/1.GO/wfs1KOvsWT/UP/wfs1KOvsWT.up.GObp_DAG.pdf]

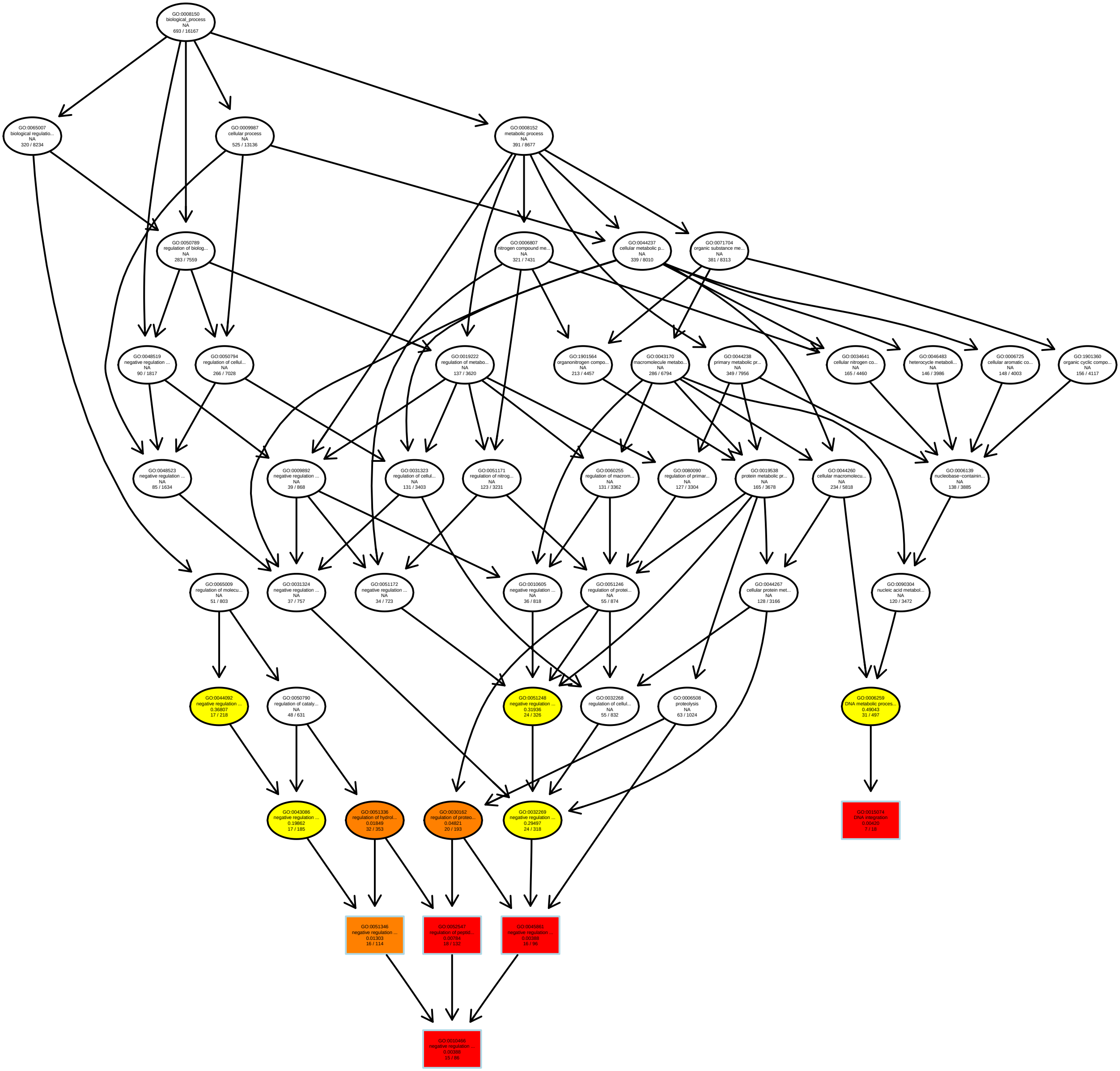

GO:0008150 biological_process NA 693 / 16167
GO:0065007 biological regulatio... NA 320 / 8234
GO:0009987 cellular process NA 525 / 13136
GO:0008152 metabolic process NA 391 / 8677
GO:0050789 regulation of biolog... NA 283 / 7559
GO:0006807 nitrogen compound me... NA 321 / 7431
GO:0044237 cellular metabolic p... NA 339 / 8010
GO:0071704 organic substance me... NA 381 / 8313
GO:0048519 negative regulation ... NA 90 / 1817
GO:0050794 regulation of cellul... NA 266 / 7028
GO:0019222 regulation of metabo... NA 137 / 3620
GO:1901564 organonitrogen compo... NA 213 / 4457
GO:0043170 macromolecule metabo... NA 286 / 6794
GO:0044238 primary metabolic pr... NA 349 / 7956
GO:0034641 cellular nitrogen co... NA 165 / 4460
GO:0046483 heterocycle metaboli... NA 146 / 3986
GO:0006725 cellular aromatic co... NA 148 / 4003
GO:1901360 organic cyclic compo... NA 156 / 4117
GO:0048523 negative regulation ... NA 85 / 1634
GO:0009892 negative regulation ... NA 39 / 868
GO:0031323 regulation of cellul... NA 131 / 3403
GO:0051171 regulation of nitrog... NA 123 / 3231
GO:0060255 regulation of macrom... NA 131 / 3362
GO:0080090 regulation of primar... NA 127 / 3304
GO:0019538 protein metabolic pr... NA 165 / 3678
GO:0044260 cellular macromolecu... NA 234 / 5818
GO:0006139 nucleobase-containin... NA 138 / 3885
GO:0065009 regulation of molecu... NA 51 / 803
GO:0031324 negative regulation ... NA 37 / 757
GO:0051172 negative regulation ... NA 34 / 723
GO:0010605 negative regulation ... NA 36 / 818
GO:0051246 regulation of protei... NA 55 / 874
GO:0044267 cellular protein met... NA 128 / 3166
GO:0090304 nucleic acid metabol... NA 120 / 3472
GO:0044092 negative regulation ... 0.36807 17 / 218
GO:0050790 regulation of cataly... NA 48 / 631
GO:0051248 negative regulation ... 0.31936 24 / 326
GO:0032268 regulation of cellul... NA 55 / 832
GO:0006508 proteolysis NA 63 / 1024
GO:0006259 DNA metabolic proces... 0.49043 31 / 497
GO:0043086 negative regulation ... 0.19862 17 / 185
GO:0051336 regulation of hydrol... 0.01849 32 / 353
GO:0030162 regulation of proteo... 0.04821 20 / 193
GO:0032269 negative regulation ... 0.29497 24 / 318
GO:0015074 DNA integration 0.00420 7 / 18
GO:0051346 negative regulation ... 0.01303 16 / 114
GO:0052547 regulation of peptid... 0.00784 18 / 132
GO:0045861 negative regulation ... 0.00388 16 / 96
GO:0010466 negative regulation ... 0.00388 15 / 86
